# Data on investigation of potential gold mineralization sites in Tchollire and Environs (North Cameroon)

**DOI:** 10.1016/j.dib.2024.110047

**Published:** 2024-01-10

**Authors:** Quentin Marc Anaba Fotze, Marcelin Bikoro Bi-Alou, Théophile Ndougsa Mbarga, Jean Paul Sep Nlomngan, Abdoul Aboubakar, Christian Mana Bouba, Ismaila Ahmadou, Alifa Guedala, Lydie Konga, Oussena Nzie

**Affiliations:** a Centre for Geological and Mining Research, P.O. Box 333, Garoua, Cameroon; b Department of Earth Sciences, Faculty of Science, University of Maroua, P.O. Box 814, Maroua, Cameroon; c Department of Physics, Advanced Teacher's Training College, University of Yaoundé I, P.O. Box 47, Yaoundé, Cameroon

**Keywords:** Thin section observations, Spectral measurements, Hydrothermal alteration minerals, Gold mining investigation, Tchollire and environs, Cameroon

## Abstract

Fresh and altered rock samples were collected and analyzed during field and laboratory studies in Tchollire and Environs. This approach aimed at the delineation of hydrothermal alteration minerals which according to the geological and mining settings of Tchollire and Environs, may be associated with gold mineralizations sites. Field investigations were achieved during the dry season to ensure the representativeness and reliability of our samples whose collections were constrained by earlier remote sensing and geophysical studies as expressed in [1]. An optical microscope both in reflected and transmitted light was used for petrographic analyses of thin and polished sections of rock samples. Other rock samples were prepared for spectral measurements which were achieved using an analytical spectral device spectrometer. The data presented here are further interpreted and discussed in [1].

Specifications Table

**Table:** 

Subject	Earth Sciences
Specific subject area	Petrography, Geophysics
Data format	Raw, Analysed
Type of data	Table, figures, graphs
Data collection	The fieldwork campaign was carried out during the PRECASEM (Projet de Renforcement des Capacités dans le Secteur Minier au Cameroun). During this investigation, a Global Positioning System (GPS) Garmin 64S was used to register the geographic coordinates of 150 rocks. 24 rocks located in potential gold mining zones were collected for further analyses. Micro-petrographic observations were achieved from an optical microscope in reflected and transmitted light. The spectra of selected rock samples were obtained using an analytical spectral device spectrometer (ASD FieldSpec 3). The mineral spectra were traced and processed for correspondence with reference mineral using The Spectral Geologist (TSG) software.
Data source location	The data were collected in Tchollire and Environs (North Cameroon) as reported in [1]. Data concerning the geographic coordinates of collected samples are displayed in Table 1. They are also available in the data repository.
Data accessibility	The analysed data are presented within this article. The Raw data are shared in the data repository and consist of one folder and two Excel files Repository name: Quentin dataset Data identification number: 10.17632/p99227dppc.2 Direct URL to data: https://data.mendeley.com/datasets/p99227dppc/2
Related research article	Q.M. Anaba Fotze, M. Bikoro Bi-Alou, T. Ndougsa-Mbarga, L. Bailly, J. Bernard, J. Penaye, J.P. Sep Nlomngan, A.E. Djieto Lordon, Y.B. Ketchaya, P.A. Moussango Ibohn, Integrating Aster 07XT, Landsat 8, and aeromagnetic data for the delineation of potential mineralization sites in North Cameroon. Geological Journal, 57(9), (2022) 3949–3971 [1].

## 1 Value of the data

• Spectral measurements and petrographic analyses of rocks collected in high potential zones (identified from earlier remote sensing and geophysical studies) reveal that they have mostly undergone propylitic and phyllic alteration through chloritization and sericitization comprehensively. The occurrence of those hydrothermal alteration minerals is associated with gold ore deposits which are currently exploited in the area.
• The data from field investigations and petrographic analyses show that the common minerals which occur with gold-bearing quartz veins in the study area include iron oxides (hematite, magnetite), pyrite, chalcopyrite, arsenopyrite, galena and quartz.
• The data from rock spectra analyses show that the common alteration minerals connected with highly potential gold prospects in the study area are kaolinite, montmorillonite, muscovite, sericite, calcite, chlorite, and epidote.
• The data provide insights on the knowledge of the geological and gold mining contexts of the study area, which could help researchers and mining companies in their prospection or exploration processes.
• The data confirm the reliability and applicability of remote sensing and aeromagnetic techniques in the prospection of hydrothermal alteration minerals linked to gold mineralizations in the study area.

## 2 Background

The National Development Strategy-Cameroon 2030 (NDS30) (available at http://onsp.minsante.cm/fr/publication/262/national-development-strategy-2020–2030) aims to make Cameroon an emerging country with respects to the sustainable development goals of Vision 2035. To this end, one of the objectives of the NDS30 is to promote and establish favourable conditions to economic growth through the exploration, discovery, and exploitation of new mining sites all over the country. Accordingly, the processing of both remotely sensed images and aeromagnetic data were carried out in Tchollire and Environs. This study allowed the delineation of potential gold mining sites whose groundtruthing was achieved from field and laboratory studies [1]. Hence, the data presented here represent outcomes from the methodological approaches used during field and laboratory studies. In addition, raw data from the analytical studies of selected samples are provided. The dataset addresses detection, location, classification and decision-making for prospective mining studies occurring in Tchollire and Environs.

## 3 Data Description

The data compiled in this article were obtained from field investigations and laboratory analyses. The study was focused on ground truthing from earlier remote sensing and geophysical results [1]. Hence, ground truthing involved confirmation of those results through the identification of hydrothermally altered minerals associated with potential gold mineralizations in Tchollire and Environs (North Cameroon).

**Fig. 1:**
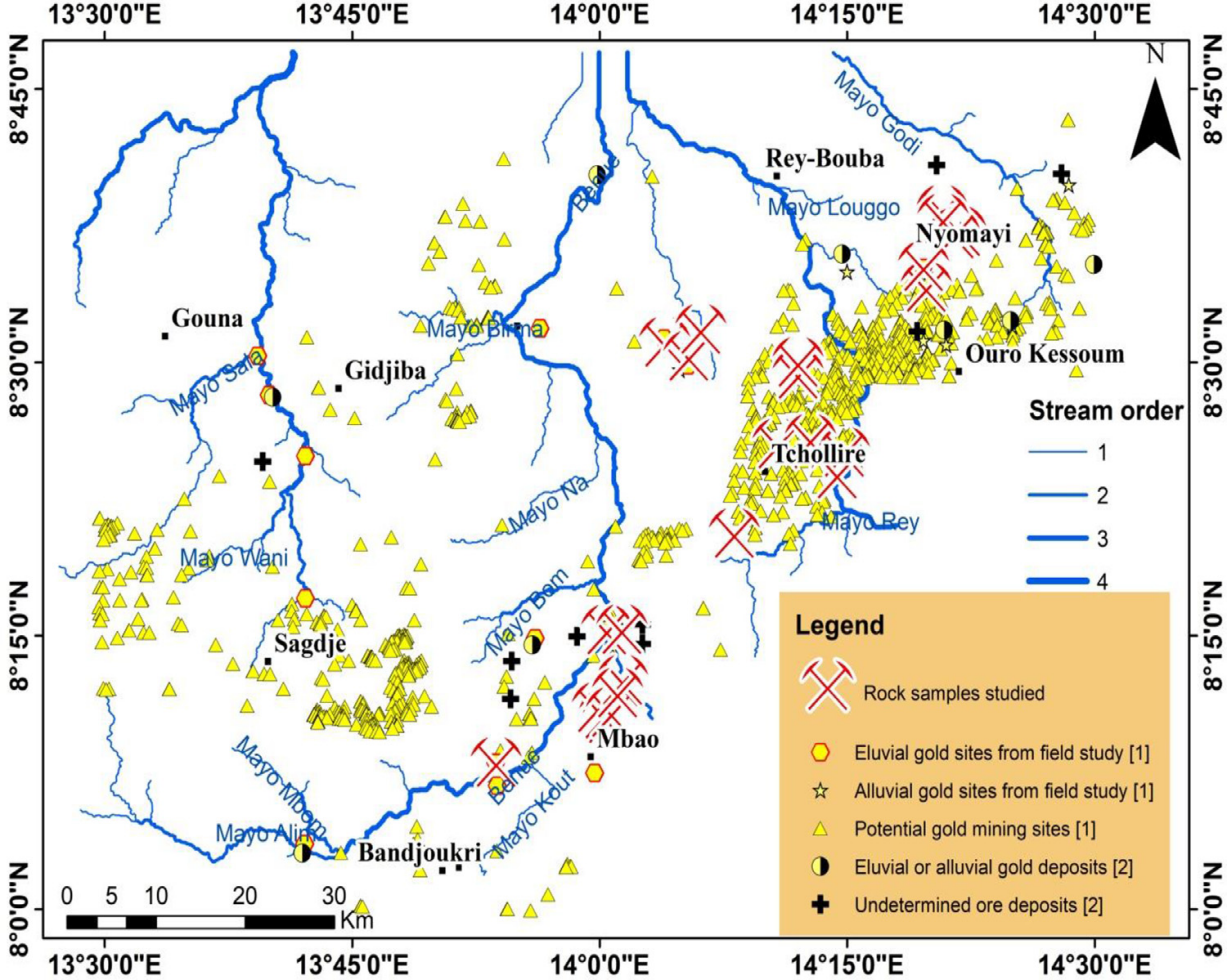
Spatial distribution of studied rock samples, overlaid on known or potential ore deposits recorded from previous studies in the study area.

**Fig. 2:**
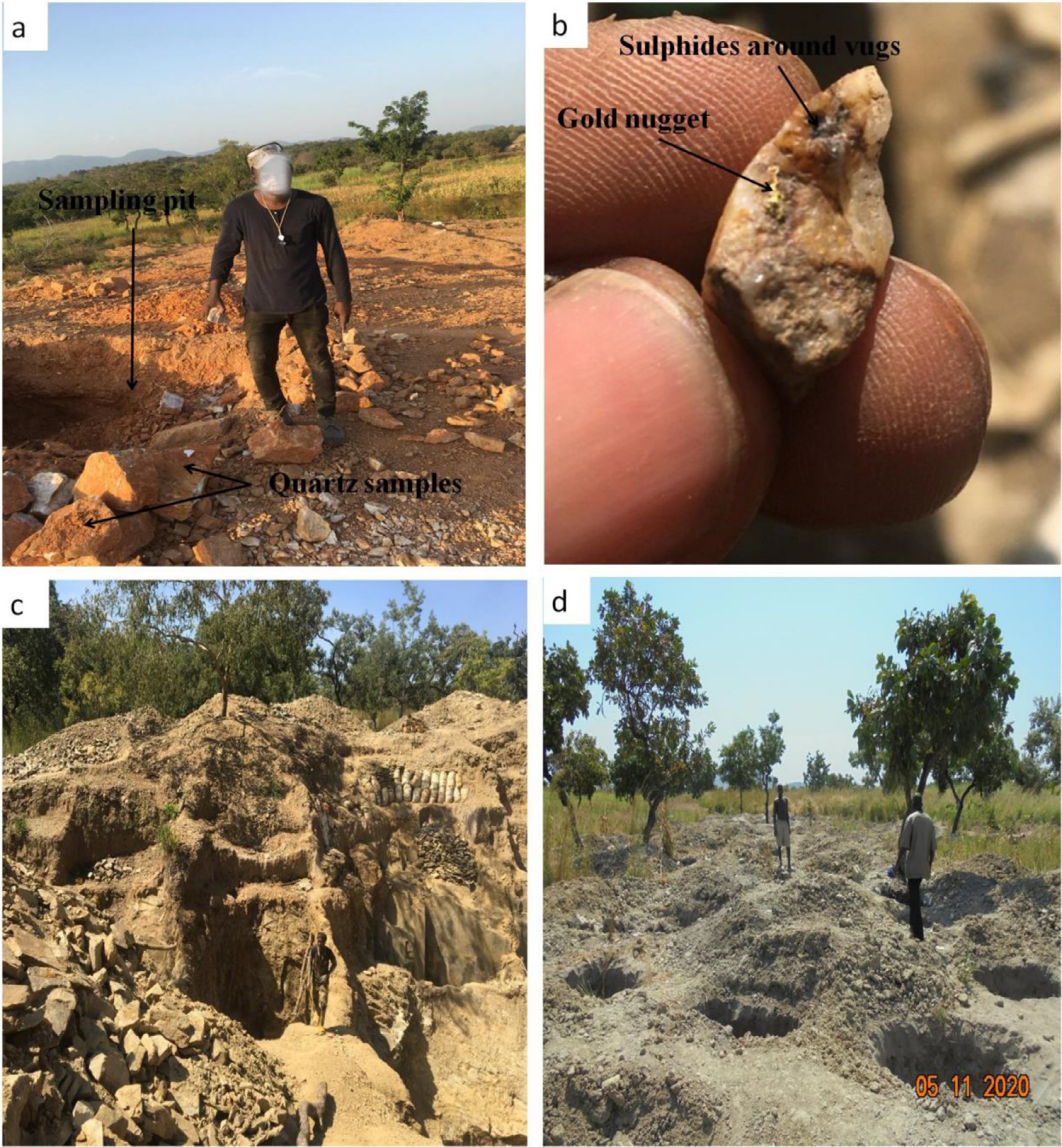
Field photographs of gold mining sites and gold-bearing quartz hand specimen: a) site of artisanal exploitation of gold mineralization displaying quartz sampling pit in Bougouma with b) gold nugget in a quartz sample depicting sulfides around the vugs; c) Primary gold artisanal exploitation site showing sampling pits in Bandjoukri locality; d) active alluvial gold exploitation site in Vaimba locality.

**Fig. 3:**
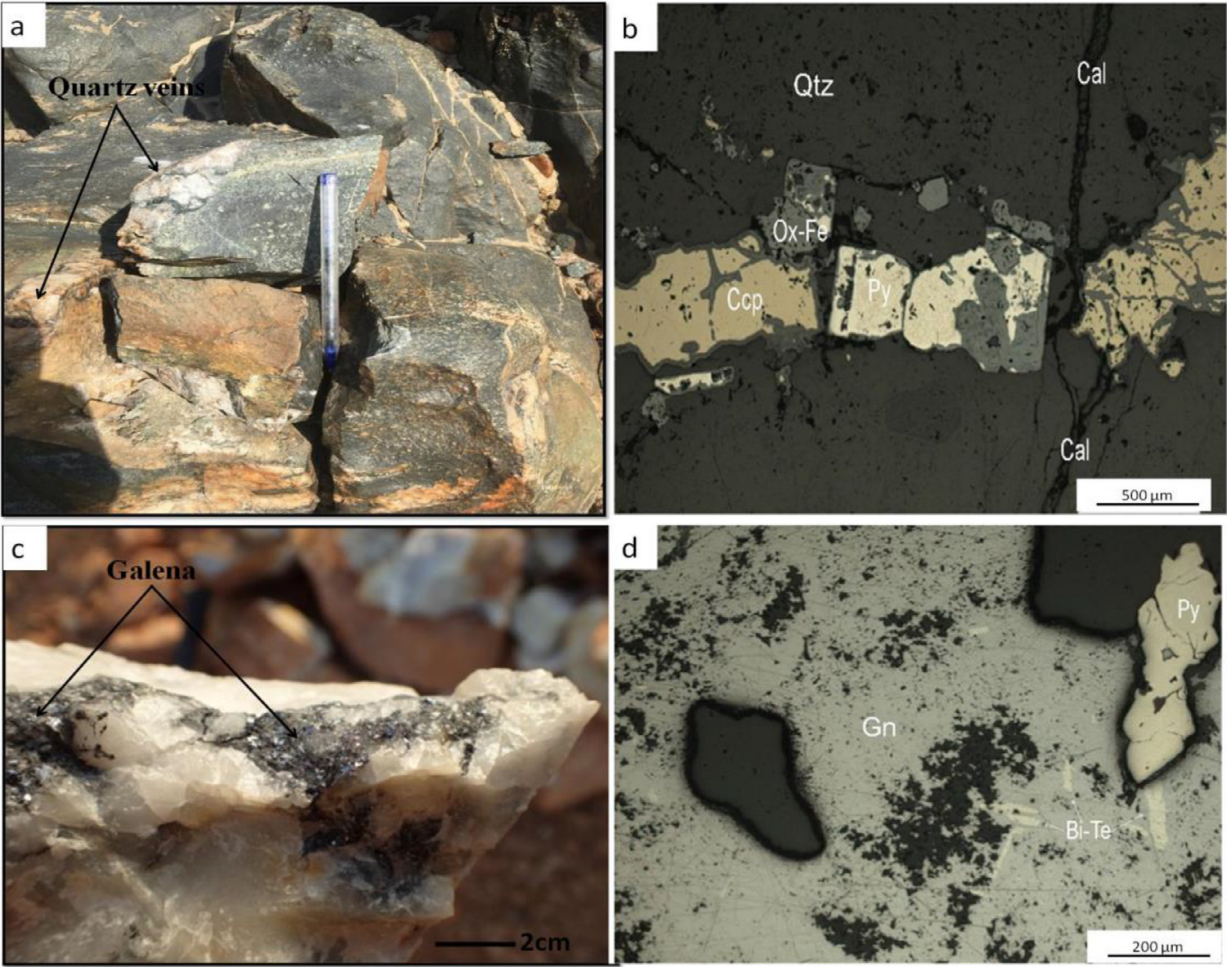
Field photographs (a, c) and photomicrographs (under analysed reflected light; b,d) of quartz veins hand specimens: a) (REY 5077) quartz veins crosscutting epidote-bearing metabasite and its b) thin section showing a quartz fracture filled with partially altered sulphides; c) (REY 5032) hand specimen of quartz associated with galena collected at Bougouma locality and its d) thin section depicting a matrix of galena partially filled with pyrite and tellurides. Mineral abbreviations: Bi-Te (Bismuth telluride); Cal (calcite); Ccp (chalcopyrite); Gn (galena); Ox-Fe (iron oxide); Py (pyrite); Qtz (quartz).

**Fig. 4:**
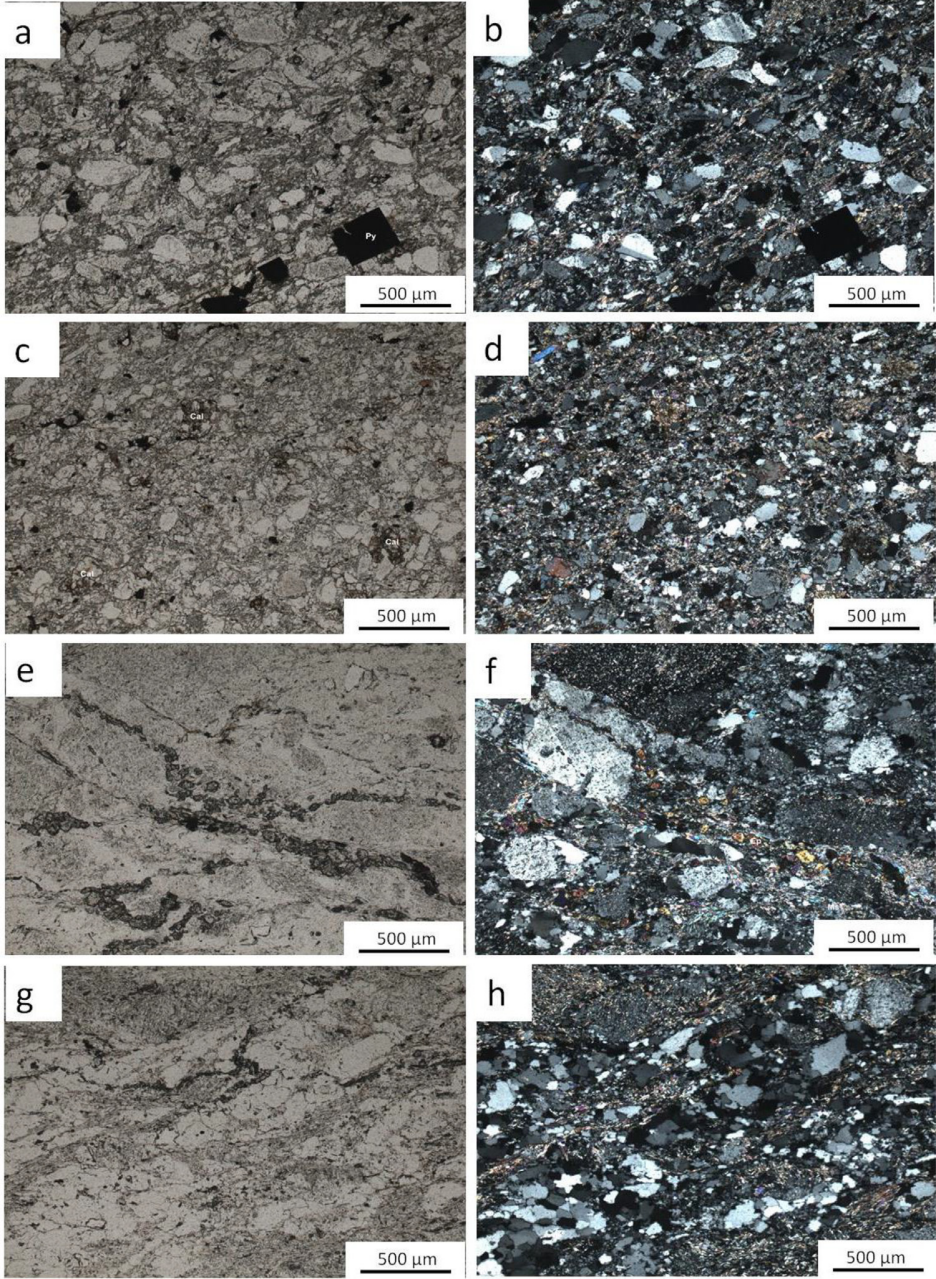
Photomicrographs (under both analysed transmitted light and polarized analysed transmitted light) of a-b) (MIN 5017) quartz-biotite-feldspar greywacke displaying alteration of feldspar into sericite (Phyllic alteration); pyrite are the opaque minerals; c-d) (MIN 5020) calcite bearing arkosic sandstone or possible volcano-sedimentary rock e-f) (MIN 5024) muscovite-sericite-epidote granite; g-h) (MIN 5025) muscovite-epidote-biotite mylonitized granite; late chloritization of biotite is remarkable.

**Fig. 5:**
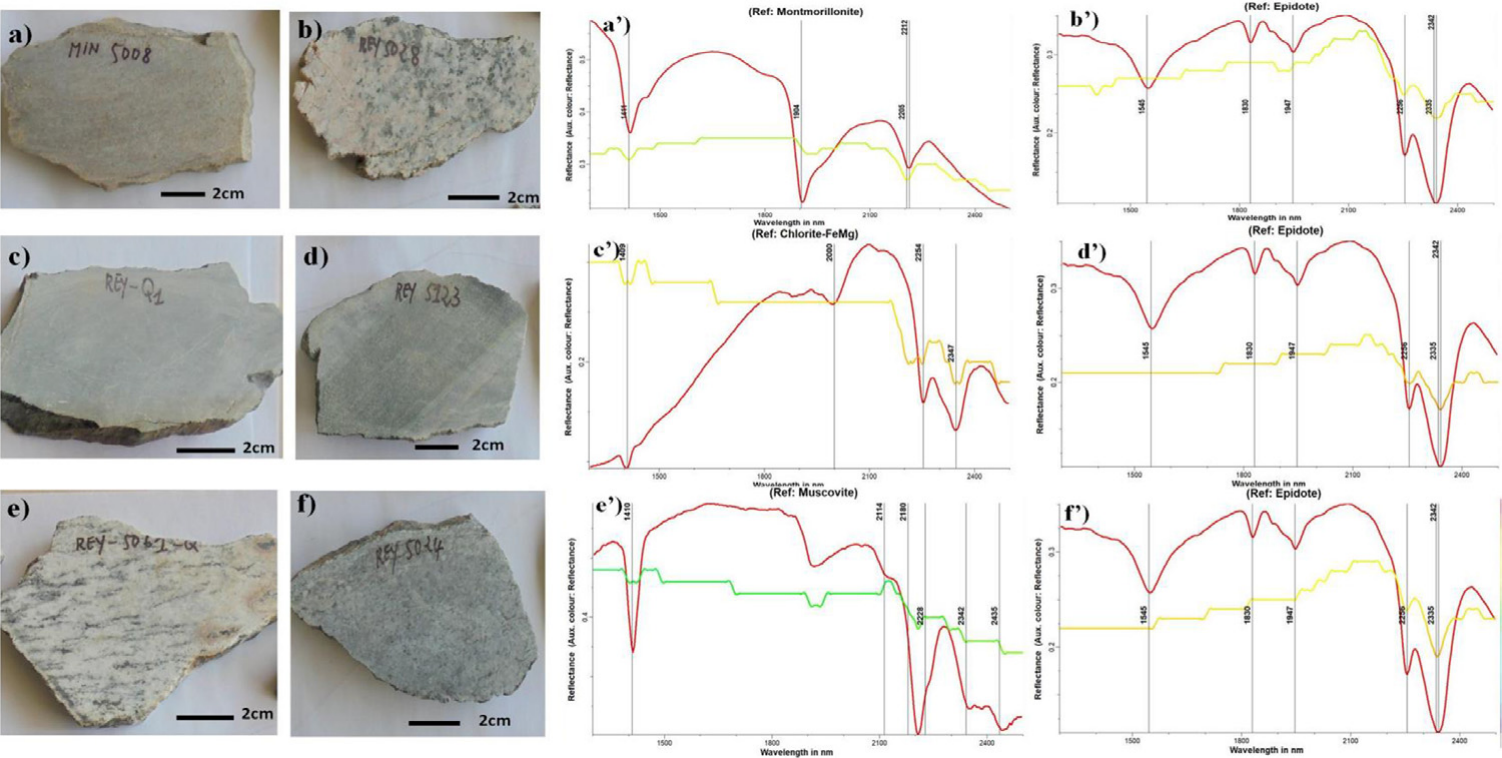
Field photographs of some rock samples and their respective spectral signatures (displayed from a’-f’ respectively) of major mineral: a) quartz-biotite-feldsapth bearing greywacke. Reaction with HCl. Probable carbonation from hydrothermal processes; b) granite from dyke; c) Metabasite: host rock of gold-bearing quartz veins; d) epidote-bearing gneiss; e) magnetite-bearing gneiss; f) gneiss crosscut by epidotitic veins. These minerals are linked to argillic, phyllic and propylitic alteration processes responsible for gold occurrence in Tchollire and Environs.

Raw data are available in the data repository as a main folder named “data for mining investigations”. The main folder contains one folder named “pictures of rock samples used for sample analyses”, two Excel files and a JPG file. The Excel file “Rock samples description” has two sheets. The sheet “data” provides the geographic coordinates (longitude and Latitude) and a brief description of the collected rocks which is displayed in this paper as Table 1; the sheet “Legend” gives the significance of some particular samples codes. The other Excel file “Rocks spectral measurements” displays two columns: the first column shows the wavelength while the second column provides the reflectance obtained from respective wavelengths. The JPG file named “thin sections” depicts the studied thin sections as displayed in Fig. 4. Other raw and analysed data are presented in this article. The spatial distribution of the analysed rock samples whose coordinates are displayed in Table 1 are depicted in Fig. 1, associated with known and potential gold deposits [1,2]. Fig. 2 depicts field photographs of primary and secondary gold mining sites and gold-bearing quartz hand specimen in the study area. Field photographs and photomicrographs (under analysed reflected light) of quartz veins samples collected in potential gold mining sites [1] are presented in Fig. 3. Fig. 4 displays Photomicrographs (under both analysed transmitted light and polarized analysed transmitted light) of some rocks collected in potential gold mining sites as well. Field photographs of rock samples and their respective spectral signatures are shown in Fig. 5.

**Table 1:** Rock samples locations and descriptions.

Sample Code	Longitude	Latitude	Lithology
MIN5008	14.327150	8.587880	Quartz-biotite-feldspar bearing greywacke. Reaction with HCl. Probable carbonation from hydrothermal processes
MIN5011	14.327380	8.586570	Argilitized and deformed quartzo-feldspathic rock. Reaction with HCl
MIN5017	14.363660	8.620200	Quartz-biotite-feldspar bearing greywacke. Reaction with HCl. (thin section)
MIN 5024	14.346630	8.631590	Muscovite-sericite-epidote bearing granite (thin section)
MIN5025	14.345530	8.638250	Altered leucocratic granite with mylonitized bands. Reaction with HCl (thin section)
POL5006	13.997757	8.184484	Tonalitic gneiss crosscut by small epidotitic veins
REY Q1	13.894902	8.133248	Metabasite : host rock of gold-bearing quartz veins (primary gold exploitation site)
REY5003	14.239908	8.398293	Tonalitic gneiss
REY5005	14.248458	8.418863	Orthogneiss
REY5014	14.179339	8.424184	Basic schists
REY5018	14.213682	8.428973	Tonalitic gneiss
REY5022	14.199993	8.489577	Altered magnetite-bearing granite
REY5024	14.199910	8.500193	Gneiss crosscut by epidotitic veins
REY5028	14.102200	8.530592	Granite occurring as a dyke
REY5032	14.065102	8.518840	Quartz veins rich in Galena and sulfides in an eluvial gold site (thin section)
REY5061	14.005291	8.178855	Magnetite-bearing gneiss
REY5063	14.007712	8.173753	Granodiorite
REY5065	14.011859	8.180192	Granodiorite
REY5068	14.023036	8.209727	Ultramylonite with rare porphyroblasts
REY5073	14.017992	8.198071	Orthogneiss
REY5077	14.006203	8.255343	Metabasite affected by a propylitic alteration (epidote) around quartz veins (thin section)
REY5079	14.022389	8.255921	Amphibole bearing Gneiss (important alluvial gold exploitation site)
REY5123	14.090021	8.505978	Epidote-bearing gneiss (alluvial gold exploitation site)

## 4 Experimental design, materials and methods

### 4.1 Fieldwork techniques

The processing of Landsat 8, Aster and aeromagnetic data allowed the realization of a target map [1] which was used during the ground truthing approach. Hence, fieldwork investigations were realized in two phases scheduled from 2nd November 2020 to 25th November 2020, and from 13th March 2021 to 21st March 2021. The field techniques applied included taking the geographic coordinates of outcrops, alluvial and eluvial gold mining sites occurring in the area of interest [1]. Then, a description of the outcrops was done and representative samples were collected. The Streckeisen diagram [3] was mostly used in order to attribute a subjective lithological name to the collected samples based on their macroscopic petrography. Various rock types lying on the target map signal were sampled and labelled. The labelling of both rock samples and their respective sampling bags was preceded by the prefix “REY” followed by the corresponding number for classic samples and “MIN” for potential mining samples; the pictures of each outcrop and collected rock sample were labelled as well. The collected rock samples consisted of altered rocks, fresh rocks, and quartz veins. Some samples were selected for laboratory analyses.

### 4.2 Petrographic analyses

A thin silver of the different rock samples was cut with a diamond saw and mounted on a glass slide. A finer abrasive grit is progressively used to ground smooth the sample until it reaches 30 µm thick later on; indeed, the sample has to be thin enough for light to pass through. The preparation of thin sections was done in many steps involving:

■ Cutting a slab
■ Initial lapping of the slab
■ Adding of glass slide
■ Sectioning of the slab
■ Final lapping
■ Polishing

The first and second steps were carried out at the laboratory of BEIG3 (Bureau d'Etudes et d'Investigations Géologico-minières, Géotechnique et Géophysique) in Yaounde/Cameroon, while the other ones were achieved at the Laboratory of BRGM in Orleans, France.

The detailed descriptions of those steps are available at http://nationalpetrographic.com/a-brief-introduction-on-thin-section-preparation.html.

### 4.3 Spectral measurements

An Analytical Spectral Devices (ASD FieldSpec 3) spectrometer was used for spectral reflectance measurements of 20 rock samples, collected in Tchollire and Environs; these analyses were achieved at the laboratory of Bureau de Recherches Géologiques et Minières (BRGM) in Orleans, France. The ASD FieldSpec 3 was used to spectrally measure the collected samples from the potential gold mining sites depicted in the predictive map [1]. The computation of the reflectance of rock materials was based on a contact probe (artificial light/ halogen light source). From 350 to 2500 ƞm wavelength interval, 2191 channels are recorded by the device. Besides, spectral values are digitized to 16 bits. There were 25 samples per data value for dark and white reference measurements. The acquisition of the reflectance of the target or rock material subsequently consists in the measurement of the radiance or luminance of both a white reference and the studied target (rock material). The ratio of both measurements eliminates the atmospheric effects and provides the reflectance of the target. More details on the principle and usage of spectral measurements device ASD FieldSpec 3 are available at http://www.geo-informatie.nl/courses/grs60312/material2017/manuals/600540-jfieldspec3usermanual.pdf.

Later on, the values obtained from the spectral measurements were imported to TSG (The Spectral Geologist, https://research.csiro.au/thespectralgeologist) software. Then, the automatic drawings of rocks spectra were realized and compared with the most probable mineral composition of rocks; this was based on the specific spectral absorption properties of each mineral in the SWIR interval. This approach was suitable for the identification of the most probable hydrothermal alteration mineral or process characterizing each rock.

## Limitations

Not applicable.

## Ethics Statement

The authors have read and followed the ethical requirements for publication in Data in Brief. The current work does not involve human subjects, animal experiments, or any data collected from social media platforms.

## CRediT Author Statement

**Quentin Marc Anaba Fotze:** Conceptualization, Methodology, Software, Validation, Resources, Writing, Review & Editing. **Marcelin Bikoro Bi-Alou:** Conceptualization, Methodology, Software, Validation, Resources, Writing, Review & Editing. **Théophile Ndougsa Mbarga:** Conceptualization & Supervision. **Jean Paul Sep Nlomngan:** Review & Editing. **Abdoul Aboubakar:** Software, Validation & Resources. **Christian Mana Bouba:** Conceptualization & Methodology. **Ismaila Ahmadou:** Conceptualization, Methodology, Software, Validation & Resources. **Alifa Guedala:** Writing, Review & Editing. **Lydie Konga:** Conceptualization, Methodology, Software, Validation, Resources, Writing, Review & Editing. **Oussena Nzie:** Review.

## Data Availability

Quentin dataset (Original data) (Mendeley Data)
